# Optimizing soybean variety selection for the Pan-African Trial network using factor analytic models and envirotyping

**DOI:** 10.3389/fpls.2025.1594736

**Published:** 2025-06-06

**Authors:** Maurício S. Araújo, João P. S. Pavan, André A. Stella, Bruno F. Fregonezi, Natally F. Lima, Erica P. Leles, Michelle F. Santos, Peter Goldsmith, Godfree Chigeza, Brian W. Diers, José B. Pinheiro

**Affiliations:** ^1^ Genetics Diversity and Breeding Laboratory, Department of Genetics, University of São Paulo, Piracicaba, São Paulo, Brazil; ^2^ Allogamous Plant Breeding Laboratory, Department of Genetics, University of São Paulo, Piracicaba, São Paulo, Brazil; ^3^ Feed the Future Innovation Lab, University of Illinois Urbana-Champaign, United States Agency for International Development (USAID), Washington, DC, United States; ^4^ International Institute of Tropical Agriculture, Consultative Group on International Agricultural Research (CGIAR), Ibadan, Oyo, Nigeria

**Keywords:** *Glycine max*, linear mixed models, environmental data, adaptation, stability

## Abstract

Soybean is a global food and industrial crop, however, climate change significantly affects its grain yield. Therefore, the selection of varieties with high adaptation to target population of environments is imperative in Sub-Saharan Africa. This study aimed to identify soybean varieties with high overall performance and stability using multi-environment trial data from the Pan-African Soybean Trial Network. Additionally, we sought to determine the environmental factors influencing yield through envirotyping tools. In two South-Eastern African countries, a total of 169 soybean varieties were evaluated across 83 environments in 19 locations in Malawi (47 trials) and 14 locations in Zambia (36 trials). The trials followed a randomized complete block design with three replications. Data for 37 environmental features were obtained from NASA POWER and SoilGrids. We fitted factor analytic models (FA) to estimate genotype adaptation across environments. Additionally, we applied an environmental kernel approach and the XGBoost method to assess the number of mega-environments. The FA model with four factors provided the best fit, explaining 82.44% and 81.95% of the variance and the average semi-variance ratio (ASVR), respectively. Approximately, 59.6% of the genotype-by-environment interaction were crossover. Varieties V025, V035, and V158 exhibited high yield potential and reliability but displayed moderate stability. Three mega-environments were identified, with growing degree days, mean temperature, and photosynthetically active radiation use efficiency being the most associated features for soybean grain yield. To enhance the identification of variety adaptation in these environments, integrating machine learning models with crop growth modeling is essential to assess associations between environmental features and soybean yield.

## 1 Introduction

Soybean (*Glycine max* L.) is a commodity crop of great global importance (Mishra et al., 2024). Its grains are widely utilized in agro-industry, primarily for oil production, high-protein food products, and animal feed formulation (Zhi et al., 2020). Its nutritional composition is determined by proteins, oil, carbohydrates, isoflavones, and minerals. However, population growth and the ever increasing demand for protein sources, both for human consumption and animal feed, highlights the need to expand global soybean production (Messina, 2022). In this context, improving production efficiency in new agricultural frontiers through the development of more adapted varieties becomes essential to ensure food security for future generations. In light of that, genetic improvement programs have focused on developing highyielding varieties with resistance to pests and diseases, as well as broad adaptation to target environmental conditions (Favoretto et al., 2025). These advancements have been driven by the optimization of breeding strategies and the adoption of effective agricultural practices (Carciochi et al., 2019).

Plant breeders rely on multi-environment trials (METs) to evaluate genotype performance across diverse conditions, representing the target population of environments (TPE) and assessing genotype adaptation to specific or broad environments (Poupon et al., 2023; Malosetti et al., 2016; Costa-Neto et al., 2023; Vitale et al., 2024). When crossover interactions occur, genotype rankings vary across environments (Fehr, 1987; Cooper and Delacy, 1994), and neglecting genotype-by-environment (G×E) interaction can introduce some bias and reduce selection efficiency (van Eeuwijk et al., 2016). To quantify G×E interaction, various methods have been explored, each with distinct assumptions and applications. These include analysis of variance (Plaisted and Peterson, 1959; Shukla, 1972), regression models (Finlay and Wilkinson, 1963; Eberhart and Russell, 1966), non-parametric approaches (Lin and Binns, 1998), multiplicative models such as GGE Biplot (Yan et al., 2000) and AMMI (Gauch and Zobel, 1997; Gauch, 2008), linear mixed models (Henderson, 1949, 1950), factor analytic (FA) models — which are extensions of linear mixed models — (Piepho, 1997a, b; Smith et al., 2001b), and Bayesian approaches (Cotes et al., 2006), all widely applied in plant breeding.

Factor analytic (FA) models are a specific class of linear mixed models (LMMs) that are particularly robust in handling diverse data structures, especially unbalanced data. As a parsimonious approximation of the unstructured model, they indirectly construct the full genetic covariance structure, accounting for heterogeneous variances and covariances. This capability allows for the exploration of genetic covariance between environments or traits, making FA models well-suited for METs. Their effectiveness stems from dimensionality reduction through latent variables, known as factors (Smith et al., 2001b; Piepho, 1998). Additionally, as linear mixed models, they facilitate the inclusion of relatedness information, whether genomic (marker-based) or ancestral (pedigree) (Smith et al., 2005). Building on these principles, Smith and Cullis (2018) introduced the Factor Analytic Selection Tools (FAST), which incorporate parameters for assessing overall performance (OP) and stability via Root Mean Square Deviation (RMSD). These metrics enhance breeders’ decision-making by providing a statistically sound and comprehensive evaluation framework. Today, FA models are the benchmark for handling unbalanced MET data within the LMM framework (Tolhurst et al., 2022; Araújo et al., 2024), with recent insights by Piepho and Williams (2024) emphasizing their utility in predicting genotype performance in METs.

Beyond selecting the most appropriate statistical methods, modern plant breeding demands additional tools to enhance the predictive ability of models. Over the past decade, environmental features have emerged as valuable resources for improving predictions in METs (Xu, 2016; Resende et al., 2024). Although the integration of environmental data into genetic analyses is not a new concept (Van Eeuwijk and Elgersma, 1993; Wood, 1976), advances in hardware and data processing have enabled the use of large datasets, facilitating the incorporation of environmental features into statistical genetic models. Enviromics, a specialized field at the intersection of environmental data, statistics, and quantitative genetics, leverages plant ecophysiology to better understand how environmental factors influence plant development and the plasticity of key agronomic traits (Costa-Neto and Fritsche-Neto, 2021). In this context, envirotypes represent all sources of environmental variation affecting plant development and can serve as environmental markers in statistical genetic models, aiding in the prediction of genotypic performance in non-evaluated environments (Xu, 2016; Resende et al., 2025).

The addition of information derived from Geographic Information System (GIS) techniques into predictive models has been encouraged to improve the efficiency of breeding programs (Guarino et al., 2002). An initial effort was made by Booth (1990) aiming to indicate climatically suitable regions for the introduction of tree species at a global scale based on the environmental conditions where they were collected. Annicchiarico et al. (2006) assessed how GIS-based methodologies could aid the recommendation of durum wheat genotypes in MET, as compared to traditional methodologies. The integration of machine learning, quantitative genetics, enviromics, and GIS tools enhances the identification of environmental patterns in target environments. These resources enable the exploration of environmental homogeneity and the determination of factors influencing climatic variability, facilitating the incorporation of G×E interaction and the selection of cultivars adapted to specific conditions.

Soybean variety selection is becoming increasingly important due to its high nutritional value and economic significance in the global market. Despite its potential, generally, the adaptation of soybean varieties to Sub-Saharan African environments specifically in the South-Eastern countries of Malawi and Zambia remains largely unexplored, limiting the availability of high-performing cultivars suited to the region’s diverse agro-ecological conditions. This gap is particularly concerning given the rapid population growth and the escalating demand for affordable protein based food sources, which underscore the necessity of expanding and optimizing soybean production. Moreover, climate change exacerbates environmental variability, increasing the urgency for resilient cultivars capable of maintaining stable yields across unpredictable conditions (Sousa et al., 2019). To address this challenge, this study employs advanced selection tools to identify superior varieties with high overall performance and stability within the Pan-African Trials Network. Furthermore, the integration of envirotyping methodologies enables the exploration of associations between environmental variables and G×E interactions, facilitating the identification of specific adaptations critical for sustainable soybean production in Malawi and Zambia.

## 2 Material and methods

### 2.1 Phenotypic data and field trials

**Figure 1:**
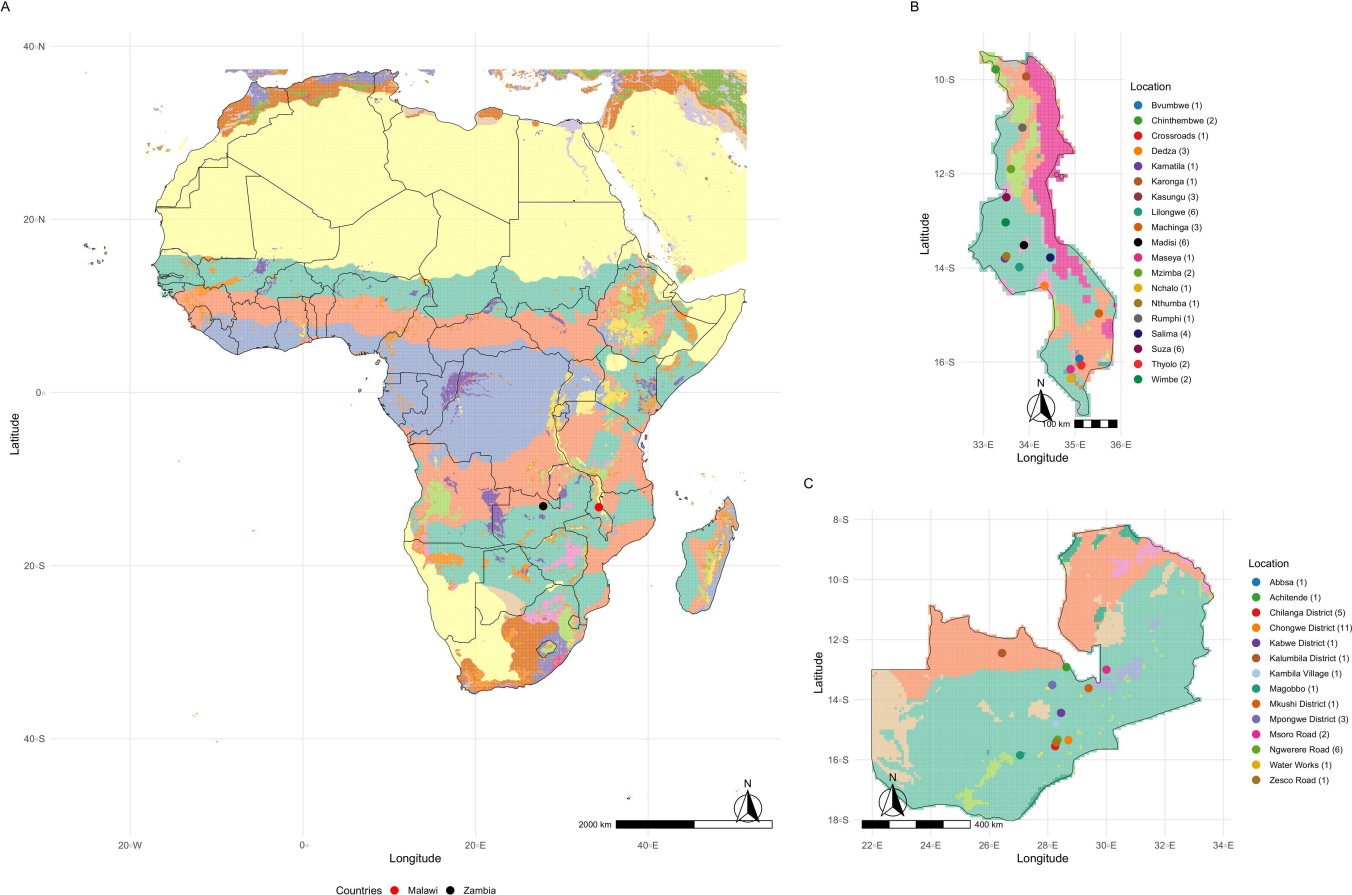
**(A)** displays the map of Africa with Agro-ecological Zones (AEZ) classified into 33 distinct categories based on climatic variables, topography, and the chemical and physical properties of the soil. Each color on the map represents a specific AEZ class. Refer to Food and Agriculture Organization of the United Nations (2025) for detailed identification of each class. The red and black points on the map highlight the countries of Malawi and Zambia, respectively. **(B)** presents the map of Malawi, highlighting its respective AEZs. The colors of the points indicate the locations where the trials were conducted, and the number in parentheses represents the number of trials carried out at each site. **(C)** shows Zambia with the distribution of trial locations, along with the number of experimental trials conducted in each region.

Soybean variety yield trials are part of the Soybean Innovation Lab (SIL). This program was established to select high-yielding varieties adapted to target population environments (TPE) in Africa, to support cultivation by smallholder farmers. This initiative led to the creation of the Pan-African Soybean Variety Trials (PATs) through partnerships with the African Agricultural Technology Foundation (AATF), the Syngenta Foundation for Sustainable Agriculture (SFSA), and the International Institute of Tropical Agriculture (IITA) (Santos, 2019). The PATs program plays a key role in identifying and disseminating varieties capable of adapting to diverse Agro-ecological conditions, thereby contributing to enhanced food security and economic growth across selected Africa countries. The African continent was divided into 33 Agro-ecological Zones (AEZs), classified according to criteria such as climatic zones (tropical, temperate, etc.), length of the growing season, soil type, and altitude, with a resolution of 5 arc-minutes (≈ 9.2 km × 9.2 km) (**Figure 1**) (Food and Agriculture Organization of the United Nations, 2025).

A total of 169 soybean varieties were evaluated over the 2017/18 to 2023/24 seasons (**Supplementary Figure S1**) in trials conducted in two South-Eastern African countries of Malawi and Zambia. In Malawi, 47 trials were conducted across 19 distinct locations, each defined as the interaction between location and season (**Figure 1B**). In Zambia, 36 environments were carried out across 14 locations (**Figure 1C**). The trials followed a randomized complete block design (RCBD) with three replications. Each plot consisted of four rows measuring five meters in length (4 × 5 m), spaced 50 cm apart, with 20 plants per row. grain yield (kg ha^−1^) was measured from the two central rows. Agronomic management practices adhered to the specific technical recommendations for soybean cultivation.

### 2.2 Envirotyping

Throughout the crop’s growing season, we collected data on 37 environmental features (**Table 1**). Each genotype’s sowing and harvesting dates were used to retrieve environment-specific variables, enabling the characterization of trial conditions and the assessment of their similarity. The environmental covariates encompassed geographic, climatic, and soil information. The climatic variables were obtained using the EnvRtype package (Costa-Neto et al., 2021), which accesses the NASA POWER database (https://power.larc.nasa.gov/) (Sparks, 2018; NasaPower, 2022). Soil attributes were retrieved from the SoilGrids database via API using the httr package for web access (Wickham, 2023) and jsonlite for JSON parsing (Ooms, 2014). Static variables such as altitude and soil properties were associated with the trial location coordinates.

**Table 1:** Summary statistics of 37 environmental features grouped into geographical, climatic, and soil-related categories.

Class	Features	ID	Unit	Min	Mean	Max
Geographical	Altitude	alt	meters (m)	70.00	1039.00	1359.00
Climatic	Mean temperature	tmean	°C	17.57	21.90	26.29
	Maximum temperature	tmax	°C	24.23	27.11	31.27
	Minimum temperature	tmin	°C	11.59	17.60	23.68
	Precipitation	prec	mm/day	0.02	5.66	11.63
	Wind speed	wsm	m/s	1.60	2.27	4.07
	Relative humidity	rhm	%	49.96	77.31	88.06
	Dew point temperature	tmdew	°C	9.19	17.13	21.43
	Longwave radiation	lw	MJ/m^2^/day	28.96	32.41	35.77
	Shortwave radiation	sw	MJ/m^2^/day	16.79	20.08	22.89
	Growing degree days	gdd	°C d−1	10.33	14.36	18.38
	Radiation use efficiency	fue	–	0.47	0.65	0.84
	Temperature range	tmrange	°C day	4.67	9.52	13.94
	Vapor pressure deficit	vpd	kPa	0.43	0.84	1.81
	Slope of vapor pressure curve	spv	kPa/°C	0.13	0.17	0.20
	Potential evapotranspiration	etp	mm/day	7.63	9.02	10.38
	Precipitation deficit	petp	mm/day	-9.29	-3.36	3.19
	Total precipitation	totprec	mm	2.69	772.73	1451.87
	Average precipitation	aveprec	mm/day	0.02	5.66	11.63
	Evapotranspiration tolerance	etptol	mm	847.00	1300.00	2185.00
	Water balance	watbal	mm	-1802.70	-526.80	376.20
Soil	Bulk density of fine earth	bdod	kg/m^3^	120.00	142.60	155.00
	Cation exchange capacity	cec	cmol/kg	60.00	89.28	142.00
	Coarse fragments volume	cfvo	%	2.00	23.62	67.00
	Clay content	clay	%	105.00	205.40	436.00
	Nitrogen content	nit	g/kg	84.00	118.00	170.00
	Organic carbon density	ocd	kg/m^3^	165.00	210.90	257.00
	Soil pH (H_2_O)	phh2o	–	54.00	61.19	64.00
	Sand content	sand	%	336.00	653.10	811.00
	Silt content	silt	%	63.00	141.50	257.00
	Soil organic carbon	soc	g/kg	115.00	153.70	206.00
	Soil water content at 10 kPa	wv0010	–	249.00	308.30	383.00
	Soil water content at 33 kPa	wv0033	–	184.00	230.90	331.00
	Soil water content at 1500 kPa	wv1500	–	68.00	104.20	199.00
	Soil temperature	tsoil	°C	226.17	253.46	292.83
	Temperature seasonality	sts	°C	86.10	155.20	255.70
	Isothermality	iso	–	-84.60	13.67	30.70
	Mean diurnal range	mdr	–	-2.00	1.17	2.40

Data were collected from soybean varieties evaluated in Malawi and Zambia during the 2017–2024 seasons through the Pan-African Trials Network. Climatic features were obtained from NASA POWER, and soil variables from SoilGrids.

Prior to kernel construction, we applied quality control filters to remove missing or inconsistent values and standardized all continuous variables using Z-score normalization to ensure comparability across different measurement scales (Equation 1):


(1) $$Z_{ij}=\frac{x_{ij}-{\bar{x}}_{\cdot j}}{s_{\cdot j}}$$

where ${\bar{x}}_{\cdot j}$ and $s_{\cdot j}$ denote the mean and standard deviation, respectively, of the *j*-th variable across all locations.

To reduce multicollinearity, we examined the Pearson correlation matrix and flagged variable pairs with correlation coefficients. Redundant variables were removed based on domain knowledge and exploratory principal component analysis (PCA), which was implemented using the factoextra version 1.0.7 package (Kassambara and Mundt, 2016).

The final environment-by-variable matrix **W** was then used to compute the enviromic similarity kernel *KE* as described in Equation 2.


(2) $$K_{E}=\frac{WW^{⊤}}{\text{trace}\left(WW^{⊤}\right)/n}$$

where $W^{⊤}$ is the transpose of **W**, and *n* is the number of environments. This standardization ensures unit trace, allowing comparability across analyses and interpretation of diagonal elements as average similarities. The matrix **W** contains standardized environmental covariates (e.g., climatic and soil variables), with rows representing environments (location-by-year combinations) and columns corresponding to environmental descriptors.

#### 2.2.1 Identification of mega-environments

Initially, environments were grouped into mega-environments based on an enviromic similarity matrix, denoted as the enviromic kernel (*KE*). This matrix integrated 37 environmental covariates and grain yield. Hierarchical clustering was applied using the Unweighted Pair Group Method with Arithmetic Mean (UPGMA) algorithm (Sokal and Michener, 1958). The optimal number of clusters was defined using the Elbow method, and the most influential covariates were explored via principal component analysis (PCA) (Pearson, 1901). To prevent methodological circularity, the dataset was randomly split into training (70%) and test (30%) subsets prior to unsupervised learning. PCA and K-means clustering were applied exclusively to the training subset, and the resulting cluster assignments were used as categorical labels for model training.

Classification was performed using the XGBoost (*Extreme Gradient Boosting*) algorithm (Chen and Guestrin, 2016), implemented via the xgboost package. The model was configured for multi-class classification (multi:softmax) and trained using the first three principal components. The hyperparameters used were: tree depth of 6, learning rate (*η*) of 0.3, and 100 boosting iterations. The objective function minimized by the algorithm included both the predictive loss and regularization terms, and is expressed in Equation 3:


(3) $$ℒ\left(\theta \right)=\sum_{i=1}^{N}\ell \left(y_{i},{\hat{y}}_{i}^{\left(t\right)}\right)+\sum_{t=1}^{T}\text{Ω}\left(f_{t}\right),$$

where ℓ denotes the multinomial log-loss function, and the regularization term $\text{Ω}\left(f_{t}\right)$ for each tree $f_{t}$ is defined in Equation 4:


(4) $$\text{Ω}\left(f_{t}\right)=\gamma T+\frac{1}{2}\lambda \sum_{j=1}^{T}w_{j}^{2},$$

in which *T* is the number of leaves, *w_j_* is the score on leaf *j*, *γ* is the complexity penalty for the number of leaves, and *λ* controls the L2 regularization on leaf weights. All analyses were performed in R (version 4.3.1) using the following packages: cluster (Maechler et al., 2019), caret (Kuhn et al., 2020), xgboost (Chen et al., 2022), and dendextend (Galili, 2015).

To explore the relationship between environmental variables and grain yield, we fitted a multiple linear regression model using the adjusted mean yield for each environment as the response variable. The model is specified in Equation 5:


(5) $$y=\mu +\sum_{i=1}^{t}{\text{β}}_{i}X_{i}+e$$

where **y** represents the adjusted mean yield in each environment; *µ* is the intercept of the model, corresponding to the overall mean yield; ${\text{β}}_{i}$ denotes the coefficient associated with the *i*-th environmental variable; $X_{i}$ corresponds to the value of the *i*-th environmental feature; **e** is the random error term, assumed to follow a normal distribution with zero mean and constant variance. Adjusted means used as the response variable were obtained by fitting separate linear mixed models for each environment, in which genotype was included as a fixed effect and replication as a random effect. From these models, empirical best linear unbiased estimates (eBLUEs) of genotype means were extracted. Subsequently, the mean of the eBLUEs within each environment was calculated and used as the environment-level adjusted mean in the subsequent analyses.

### 2.3 Statistic analysis

We analyzed the phenotypic data using the linear mixed-effects model described by Henderson (1949) and Henderson (1950). Estimation of variance components was performed using the residual maximum likelihood (REML) method (Patterson and Thompson, 1971). The model was implemented using the ASReml-R package (version 4.1.2) (Butler et al., 2018) within the R software environment (R Core Team, 2022). Prior to model fitting, we assessed the validity of key model assumptions through standard residual diagnostics. The normality of residuals was evaluated using quantile–quantile (Q-Q) plots, as recommended by Kozak and Piepho (2018). Residual independence was assumed, and heteroscedasticity across environments was addressed by specifying a diagonal residual covariance matrix, allowing each environment to have its ϵown residual variance. The applied model follows Equation 6.


(6) $$y=\mu 1_{\text{n}}+X_{1}s+X_{2}b+Z_{1}g+Є$$

In which $y^{\left(n\times 1\right)}$ is the vector of phenotypic data across t environments, where $n=\sum_{j=1}^{t}n_{j}$ , and $n_{j}$ is the number of observations in each environment j ; μ is the model intercept; $s^{\left(t\times 1\right)}$ is the vector of fixed effects for environments; $b^{\left(b\times 1\right)}$ is the vector of fixed effects for the blocks, where $b=\sum_{j=1}^{t}b_{j}$ and $b_{j}$ is the number of blocks within environment $j;\;g^{\left(v\times 1\right)}$ is the vector of random effects for the v genotypes evaluated across environments, where $g∼\text{MVN}\left(0,G⊗I_{\text{v}}\right)$ . Although genotypes are conceptually common across environments, the factor analytic (FA) model implicitly nests genotypes within environments by modeling the genotype-by-environment interaction through the **G** matrix, which captures the variance–covariance structure among environments. ${Є}^{\left(n\times 1\right)}$ is the vector of residual effects, where $Є∼\text{MVN}\left(0,R⊗I_{n}\right)$ . Here, **R** is a diagonal matrix of order t , allowing for heterogeneous residual variances across environments, i.e., $R=\text{diag}\left(\sigma_{{Є}_{1}}^{2},\sigma_{{Є}_{2}}^{2},\ldots ,\sigma_{{Є}_{t}}^{2}\right)$ . $X_{1}^{\left(n\times t\right)}$ , $X_{2}^{\left(n\times b\right)}$ , and $Z_{1}^{\left(n\times v\right)}$ , represent the incidence matrices of the vectors accompanying them in the model. $1_{n}^{\left(n\times 1\right)}$ is a vector of ones; and $I_{v}$ and $I_{n}$ areidentity matrices of orders v and n , respectively.

The genotypic effect vector g , for an FA model of order

K , is then expressed in Equation 7:


(7) $$g=\left(\hat{\text{Λ}}⊗I_{v}\right)\hat{f}+\delta$$

where ${\hat{\text{Λ}}}^{\left(t\times K\right)}$ is the matrix containing the K factor loadings for each of the t environments $\left({\text{λ}}_{1},{\text{λ}}_{2},\ldots ,{\text{λ}}_{\text{t}}\right)$ , ${\hat{\text{f}}}^{\left(Kv\times 1\right)}$ is the vector containing the v factor scores of genotypes in each environment ${\left[f_{1}^{\text{T}},f_{2}^{\text{T}},\ldots ,f_{\text{v}}^{\text{T}}\right]}^{\text{T}}$ , and ${\hat{\delta }}^{\left(tv\times 1\right)}$ is the vector representing the model’s lack of fit. The joint distribution of $\hat{f}$ and $\hat{\delta }$ is given in Equation 8:


(8) $$\left(\begin{array}{c} \hat{f}\\ \hat{\delta } \end{array}\right)∼N\left[\left(\begin{array}{c} 0\\ 0 \end{array}\right),\left(\begin{array}{cc} I_{K}⊗I_{v} & 0\\ 0 & \text{Ψ}⊗I_{v} \end{array}\right)\right]$$

In which ${\text{Ψ}}^{\left(t\times t\right)}$ is the diagonal matrix of specific variances ( ${\text{Ψ}}_{1},{\text{Ψ}}_{2},\ldots ,{\text{Ψ}}_{t}$ ) for each environment, i.e., what the factors couldn’t capture.

The selection of the most parsimonious model was based on the explained variance $v_{kt}$ , which was utilized for all K factors and for each factor per environment ( k -th) (Equation 9) (Smith et al., 2015), and the average semi-variance ratio (ASVR) (Equation 10) (Piepho, 2019; Chaves et al., 2023), respectively.


(9) $$v_{k_{t}}=\frac{{\hat{\lambda }}_{k_{t}}^{{⋆}^{2}}d_{k}}{\sum_{k=1}^{K}{\hat{\lambda }}_{k_{t}}^{{⋆}^{2}}d_{k}+{\hat{\psi }}_{t}}\times 100$$

(10) $$\begin{array}{l} ASVR=\frac{\frac{2}{t\times \left(t-1\right)}\sum_{t=1}^{t-1}\sum_{t^{\prime }=t+1}^{t}\frac{1}{2}\times \left(\sum_{k=1}^{K}{\hat{\lambda }}_{k_{t}}^{{⋆}^{2}}+\sum_{k=1}^{K}{\hat{\lambda }}_{k_{t^{\prime }}}^{{⋆}^{2}}\right)-\sum_{k=1}^{K}{\hat{\lambda }}_{k_{t}}^{⋆}{\hat{\lambda }}_{k_{t^{\prime }}}^{⋆}}{\frac{2}{t\times \left(t-1\right)}\sum_{t=1}^{t-1}\sum_{t^{\prime }=t+1}^{t}\frac{1}{2}\times \left[\left(\sum_{k=1}^{K}\lambda_{k_{t}}^{{⋆}^{2}}+\psi_{t}\right)+\left(\sum_{k=1}^{K}{\hat{\lambda }}_{k_{t^{\prime }}}^{{⋆}^{2}}+\psi_{t^{\prime }}\right)\right]-\sum_{k=1}^{K}{\hat{\lambda }}_{k_{t}}^{⋆}{\hat{\lambda }}_{k_{t^{\prime }}}^{⋆}}\\ \times 100 \end{array}$$

The generalized heritability by Cullis et al. (2006) was obtained through the Equation 11:


(11) $$H^{2}=1-\left(\frac{{\bar{\nu }}_{BLUP}}{2\sigma_{g}^{2}}\right)$$

Where ${\bar{\nu }}_{BLUP}$ is the average pairwise prediction error variance, and $\sigma_{g}^{2}$ is the genotypic variance.

The coefficient of variation was calculated using Equation 12.


(12) $$CV=\frac{{\hat{\sigma }}_{e}}{\hat{\mu }}$$

Where ${\hat{\sigma }}_{e}$ is the estimated residual standard deviation, and $\hat{\mu }$ is the overall mean of each environment.

We estimated the genetic correlation between pairs of environments as described by Cullis et al. (2010), given by Equation 13:


(13) $$\rho_{g_{tt'}}=\;\frac{\sum_{k=1}^{K}\lambda_{tk}\lambda_{t'k}}{\sqrt{{\hat{\sigma }}_{gt}^{2}{\hat{\sigma }}_{gt'}^{2}}}=DGD$$

where, ${\hat{\sigma }}_{gt}^{2}$ and ${\hat{\sigma }}_{gt'}^{2}$ represent the genotypic variance components in environments t and t' respectively, while the matrix D is a diagonal matrix composed of the reciprocal square roots of the diagonal elements of matrix G .

The crosser interaction was estimated using Equation 14:


(14) $$\sigma_{ge_{rk}}^{2}=1-\frac{\sigma^{2}\left(\sqrt{\sigma_{g_{t}}^{2}}\right)}{\sigma_{ge}^{2}}$$

The variance component for the genotype-by-environment G × E interaction, denoted as $\sigma_{ge}^{2}$ , was estimated using a compound symmetry (CS) model. In this structure, the variance-covariance matrix of the genetic effects is definedas $\sigma_{g}^{2}J+\sigma_{ge}^{2}I_{j}$ , where J is a matrix of ones. The CS model was adopted following the conceptual framework proposed by Cooper and Delacy (1994), which enables the partitioning of G×E interaction into simple (related to genotypic response consistency) and crossover (due to changes in genotype ranking) components. By assuming equal genetic variances and covariances across environments, the CS structure provides a neutral and interpretable baseline, from which deviations can be attributed to crossover interaction. This approach avoids conflating model-derived correlation structures, such as those in FA models, with the theoretical decomposition of the G×E variance.

### 2.4 Factor Analytic Selection Tools

To address identifiability issues and enable biological interpretability in factor analytic (FA) models, we adopted the constraints implemented in ASReml-R (Butler et al., 2018), as described by Smith et al. (2021). Specifically, for models with more than one factor (*K* > 1), the upper triangular elements of the loading matrix **Λ** were set to zero, and the factor scores were assumed to have a diagonal covariance matrix with decreasing elements. The constrained loading matrix is denoted as Λ^∗^, and the corresponding factor scores as ***f*** ^∗^. To recover the original (rotated) parameterization while preserving the variance structure implied by the model, we performed a singular value decomposition (SVD) of **Λ** ^∗^ as follows in Equation 15:


(15) $${\text{Λ}}^{*}=UL^{1/2}V^{⊤},$$

where U and V are orthonormal matrices of dimensions t×K and K×K , respectively, and L is a diagonal matrix with singular values sorted in decreasing order. The final rotated loading matrix is then obtained as $\text{Λ}={\text{Λ}}^{*}VL^{-1/2}=U$ , and the diagonal matrix of factor variances is D=L . Accordingly, the scores f are reconstructed as $\left(L^{1/2}V^{⊤}⊗I_{v}\right)f^{*}$ , ensuring that the variance of the factors satisfies $\text{var}\left(f\right)=D⊗I_{v}$ , as required for proper modeling of the random effects in the FA structure. These constraints facilitate identifiability and maintain the interpretability of the latent dimensions while preserving the implied genetic covariance structure across environments.

To support genotype selection within the environments evaluated, we used FA Models and applied the selection tools proposed by Smith and Cullis (2018). Specifically, the overall performance (*OP_v_*) (Stefanova et al., 2009) of the *v*-th genotype was calculated using Equation 16:


(16) $$OP_{v}=\frac{1}{t}\sum_{t=1}^{T}{\hat{\lambda }}_{1t}^{*}{\tilde{f}}_{1v}^{*}$$

In the provided equations, ${\hat{\lambda }}_{1t}^{*}$ represents the rotated factor loading associated with the t -th environment for the first latent factor, and ${\tilde{f}}_{1v}^{*}$ denotes the rotated score of the v -th genotype for the first latent factor.

The remaining factors evaluate the stability parameter. The overall stability of the *v*-th genotype can be calculated by the root mean square deviation (*RMSD_v_*) using the following Equation 17:


(17) $$RMSD_{i}=\sqrt{\frac{1}{t}\sum_{t=1}^{T}{Є}_{t}^{*}}$$

In the given expressions, ${Є}_{vt}^{*}$ represents the deviation of the prediction associated with the first factor, which can be obtained as follows: ${Є}_{vt}^{*}={\tilde{\beta }}_{vt}-{\hat{\lambda }}_{1t}^{*}{\tilde{f}}_{1v}^{*}$ , where ${\tilde{\beta }}_{vt}$ is the linear combination of loadings and factor scores from all factors except the first.

The responsiveness of genotype *v* to the *k*-th factor ( $RE_{vk}$ ) was computed as shown in Equation 18:


(18) $$RE_{vk}=\left({\bar{\lambda }}_{k}^{*}-{\bar{\lambda }}_{k-}^{*}\right)f_{vk}^{*}$$

where ${\bar{\lambda }}_{k+}^{*}$ and ${\bar{\lambda }}_{k-}^{*}$ represent the mean of the positive and negative rotated loadings, respectively, associated with the k -th latent factor.

We evaluated the reliability of each genotype using Equation 19:


(19) $$R_{v}=1-\frac{PEV_{v}}{{\bar{\sigma }}_{g}^{2}}$$

In which ${\text{PEV}}_{v}$ is the prediction error variance of the *v*-th genotype, and ${\bar{\sigma }}_{g}^{2}$ is the mean genotypic variance across environments.

An ideal genotype should present both high overall performance (*OP_v_*) and low root mean square deviation (*RMSD_v_*). The ideal genotype is selected based on the construction of an index (*FAST_v_*) (Chaves et al., 2023; Cowling et al., 2023) (Equation 20):


(20) $$FAST_{v}=\left(2\times \frac{OP_{v}-\bar{O}P}{\sqrt{\sigma_{\left(OP\right)}^{2}}}-\frac{RMSD_{v}-R\bar{M}SD}{\sqrt{\sigma_{\left(RMSD\right)}^{2}}}\right)\times R_{v}$$

## 3 Results

**Figure 2:**
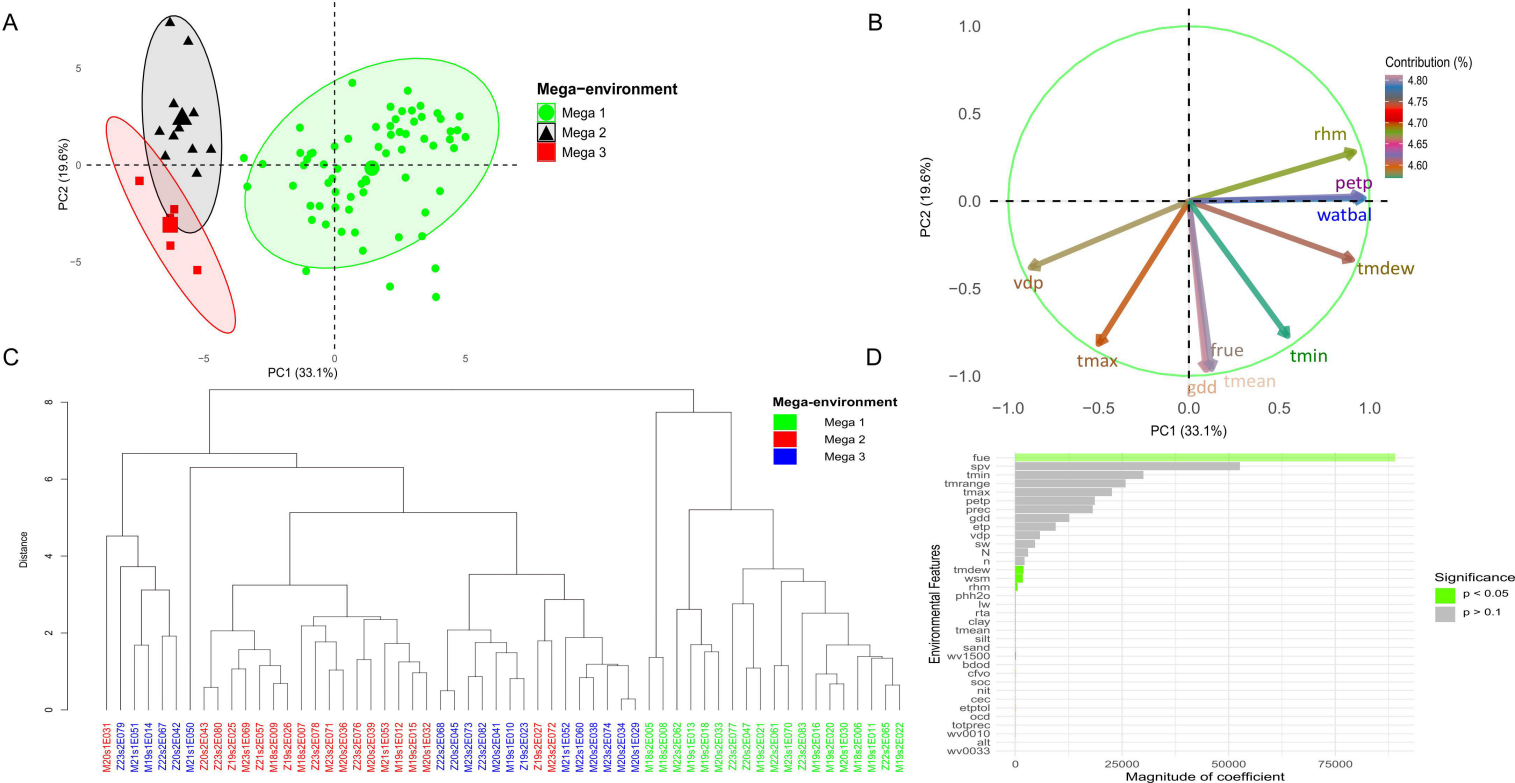
The **(A)** displays a principal component analysis (PCA) based on the environmental kernel, where the colors green, black, and red correspond to mega-environments, Mega 1, Mega 2, and Mega 3, respectively. The **(B)** highlights the environmental variables that contribute the most across all evaluation sites. The **(C)** presents a dendrogram based on the XGBoost model, used to test cluster mega-environments in Malawi and Zambia during the 2017 to 2023/24 growing seasons. Meanwhile, the **(D)** represents the variables with the greatest influence on yield performance in the trials.

Environmental kernel-based analyses incorporated climate and soil data from trials between 2017 and 2024. Principal component analysis (PCA) explained 52.7% of the total variance, with 33.1% attributed to the first principal component (PC1) and 19.6% to the second (PC2) (**Figure 2A**). Ten environmental features contributed most to climate variation among trials, with growing degree days (gdd), mean temperature (tmean), and photosynthetically active radiation use efficiency (fue) showing the strongest loadings in PC1 (**Figure 2B**). Hierarchical clustering applied to environmental similarities (based on the XGBoost model) suggested three mega-environment groups (**Figure 2C**). Regarding yield, the variables fue (radiation use efficiency), spv (seasonal precipitation variation), and tmrange (thermal amplitude) were associated with the largest regression coefficients. Additionally, fue, tmdew (mean dew point), wsm (soil moisture), and rhm (mean relative humidity) showed statistically significant associations with yield (p< 0.05) (**Figure 2D**).

The M4 model, with a factor analytic (FA) variance-covariance structure consisting of four factors (**Table 2**), exhibited the best fit for the dataset (**Supplementary Figure S2**). This selection was based on a threshold of 82.44% of the explained variance and 81.95 (%) of ASVR for the model with four factors (FA4). This criterion considered not only the explanatory capacity of the data but also the parsimony.

**Table 2:** Log-likelihood (LogL), deviance, number of parameters (Par.), explained variance (var%), and average semi-variance ratio (ASVR) for the models tested.

Model	LogL	Deviance	Par.	var (%)	ASVR (%)
M1	-57739.31	115478.62	174	37.23	34.00
M2	-57621.79	115243.58	260	65.52	62.58
M3	-57487.28	114974.56	345	77.31	77.17
**M4**	**-57395.72**	**114791.44**	**429**	**82.44**	**81.95**
M5	-57317.67	114635.34	512	87.90	87.58
M6	-57230.54	114461.08	594	93.11	92.83

The deviance (*D*) was calculated as *D* = −2 × log *L*. The model in bold is the selected one. The selection threshold was set at 80% for both explained variance (Var%) and ASVR(%), balancing goodness-of-fit and parsimony.

**Figure 3:**
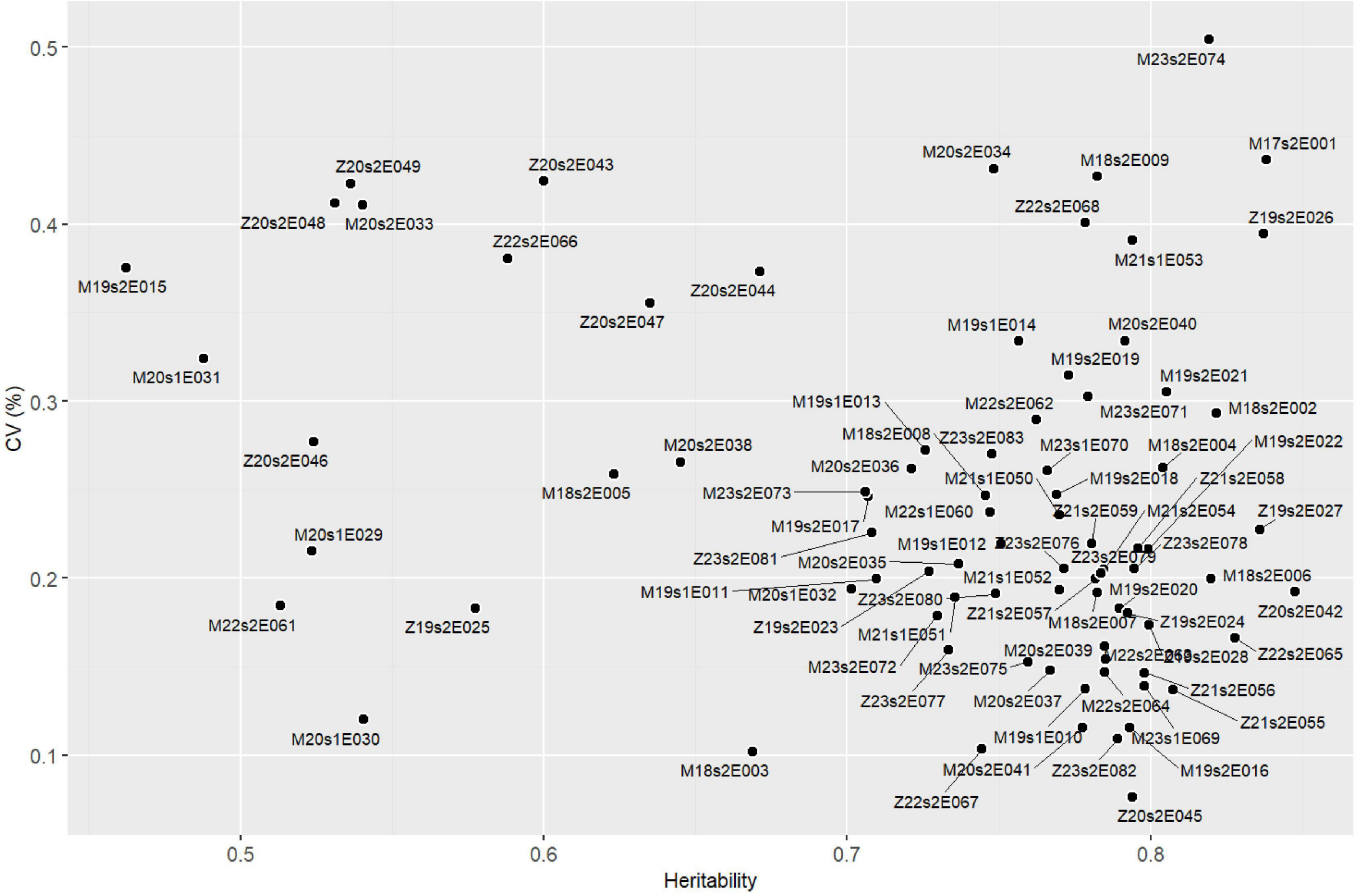
Scatterplot showing the relationship between the coefficient of variation (CV) and heritability (H^2^) across 83 soybean yield trials conducted in Malawi and Zambia. Each point represents an environment (trial), positioned according to its heritability (X-axis) and CV (Y-axis), with labels indicating the environment codes.

The Pan-Africa Trial Network demonstrated high experimental precision, with values ranging from 0.07 (M18s2E006) to 0.50 (M21s1E051). Broad-sense heritability coefficients (*H* ^2^) were also substantial, ranging from 0.46 (M19s2E015) to 0.85 (Z21s2E059) (**Figure 3**). Based on the distribution, the coefficient of variation (CV) showed a median of 0.229, with first and third quartiles of 0.183 and 0.272, respectively. Similarly, *H* ^2^ values had a median of 0.768, with Q1 = 0.710 and Q3 = 0.789 (**Supplementary Figure S3**). The average yield across the trials was 2,508.54 kg ha^−1^; however, there was considerable variation among the experiments, ranging from 523.82 kg ha^−1^ (Z19s2E027) to 4,410.92 kg ha^−1^ (M22s2E062) (**Supplementary Table S1**). Considering the two countries individually, the average yield in Malawi was 3,171.10 kg ha^−1^, while in Zambia it was 2,555.94 kg ha^−1^.

**Figure 4:**
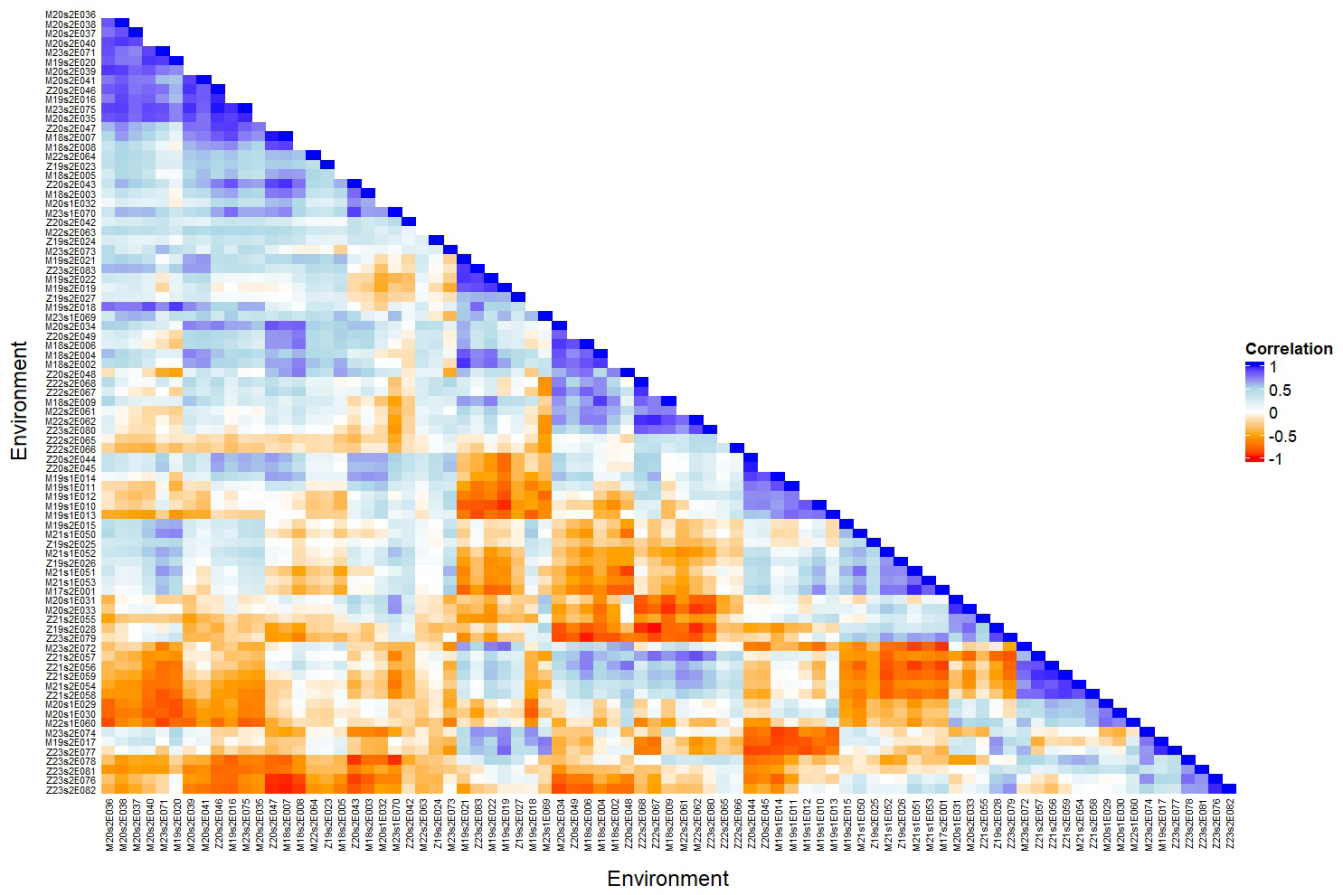
Heatmap showing pairwise genotypic correlations between environments based on the factor analytic (FA) model. Each cell represents the genetic correlation between two trials, with a color scale ranging from −1 to 1. Trial names are shown along both axes, and the figure emphasizes patterns of genetic similarity across environments. The evaluations were conducted in Malawi and Zambia from the 2017/18 to 2023/24 seasons, focusing on soybean grain yield.

**Figure 4** shows a heatmap of pairwise genetic correlations between environments based on the factor analytic (FA) model. The strongest negative correlation was observed between trials Z19s2E028 and Z22s2E067 (*r* = −0.99), indicating a strong crossover interaction. Environments Z21s2E059, Z21s2E056, and Z21s2E057 showed high variability in correlations with other trials (SD *>* 0.48), suggesting inconsistent genotype responses. In contrast, Z19s2E024 and Z22s2E065 were among the most stable environments, with the lowest standard deviation in correlations (SD< 0.23). Trials such as Z20s2E046 and M20s2E039 exhibited the highest mean correlations with other environments (mean *r >* 0.20), highlighting their potential as representative environments for genotype recommendation. These results reflect substantial heterogeneity in genotype-by-environment interactions across trials conducted in Malawi and Zambia from 2017/18 to 2023/24, emphasizing the importance of environment-specific selection.

**Figure 5:**
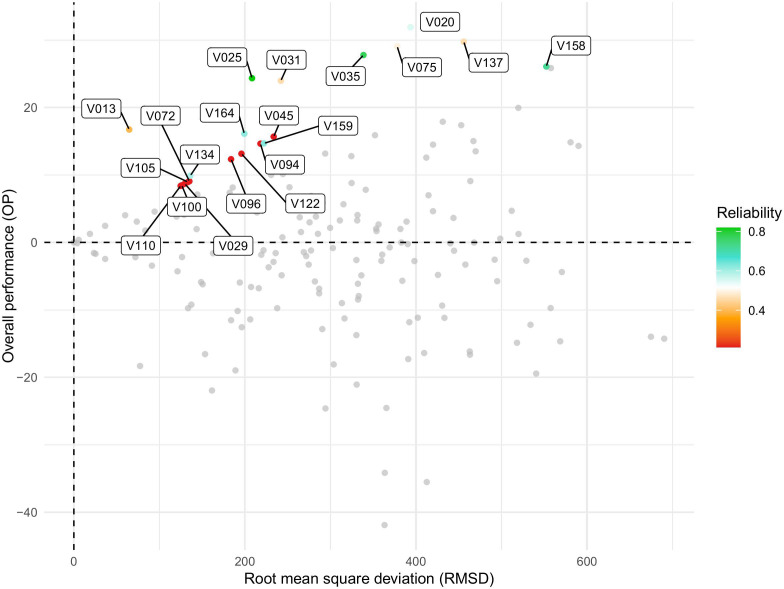
Graph showing the relationship between overall performance (OP) and stability, measured as root mean square deviation (RMSD), for soybean varieties evaluated in the Pan-African Trials Network across the 2017–2023/24 seasons. OP represents the mean performance of each genotype across environments, while RMSD quantifies the deviation from the average response, with lower values indicating higher stability. Each point corresponds to a genotype, and colors represent the reliability of the estimated performance–stability values, with the color scale ranging from red (low reliability) to green (high reliability). Axes labels and the legend have been enlarged to improve readability. This visualization summarizes results from the FAST (Factor Analytic Selection Tools) analysis.

The varieties V020, V075, V137, V158, V035, V025, and V031 exhibited the best performance, as indicated by the highest OP values (Y-axis). Regarding stability, V013 showed the best fit, with the lowest RMSD values (X-axis) according to the FAST index. Varieties V025, V035, and V158 demonstrated high yield and reliability but exhibited medium stability (**Figure 5**).

**Figure 6:**
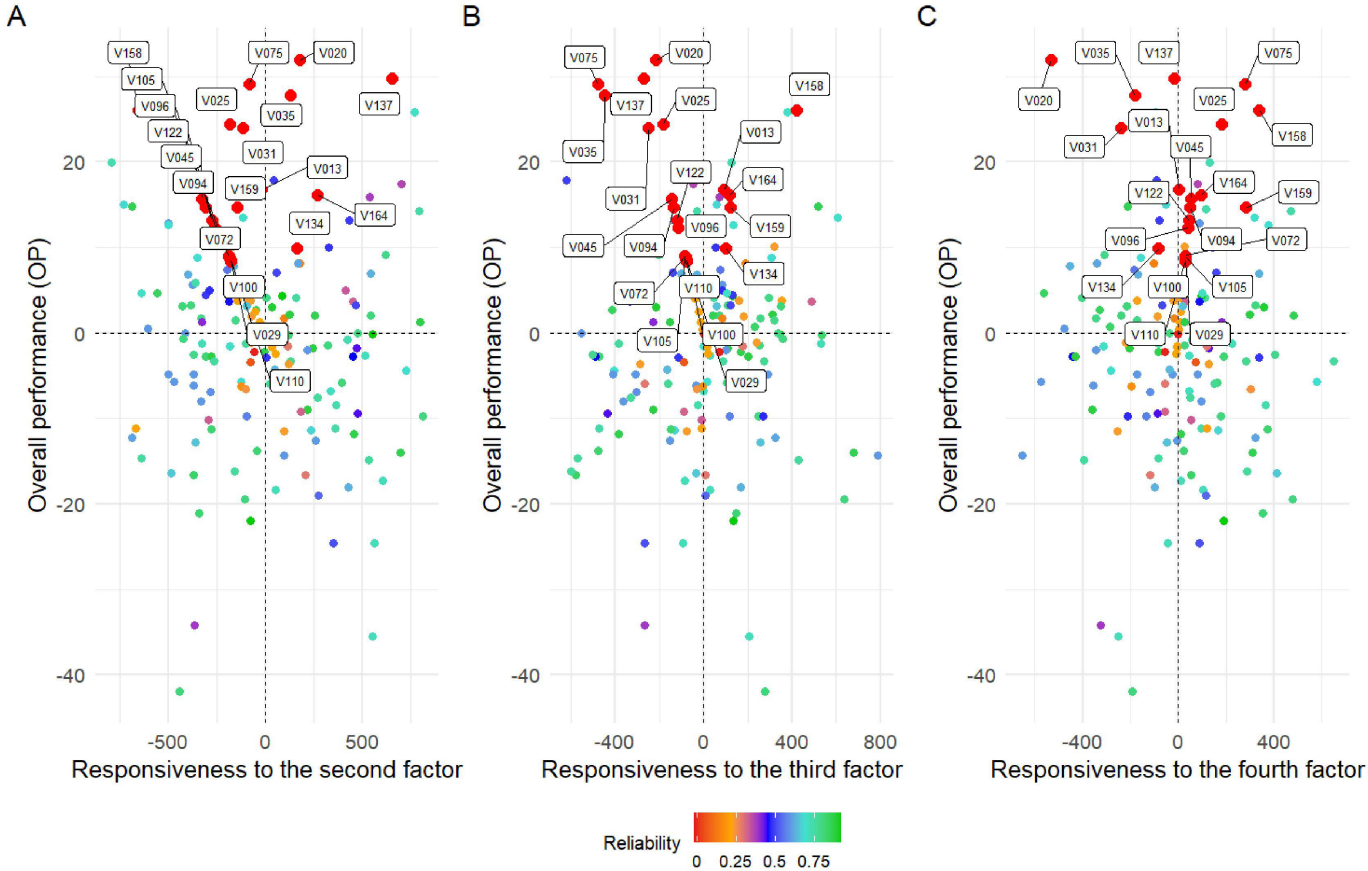
Overall performance (OP) vs. stability (RMSD) for all 169 soybean varieties from the Pan-African Trials Network. Biplot **(A)** represents responsiveness to the second factor, **(B)** to the third factor, and **(C)** to the fourth factor. Each point represents a genotype, with color indicating the reliability of its estimated performance–stability score. The color scale ranges from red (low reliability) to green (high reliability), as shown in the accompanying legend. Axes labels and the reliability legend have been enlarged to enhance readability.

**Figure 6** presents the response of the variables to the second (**Figure 6A**), third (**Figure 6B**), and fourth (**Figure 6C**) factors. Responsiveness to specific factors facilitates the identification of environmental conditions associated with the environments that contribute to these factors. In this context, varieties V075, V020, and V137 demonstrated high overall performance and stability across factors 2, 3, and 4, respectively. Conversely, genotypes exhibiting low reliability (*<* 0.4%), such as V029, V110, V100, and V105 (**Figure 5**), also consistently demonstrated the poorest overall performances across all four evaluated factors, highlighting their limited adaptability and potential. Additionally, the variety V13 maintained the best fit in terms of OP, suggesting a higher stability and suitability under the tested conditions (**Figure 6**). These findings suggest that the associated factors may reflect meaningful environmental characteristics that can be leveraged for specific adaptation.

## 4 Discussion

In this study, we applied FAST tools for selecting soybean varieties with high overall performance and stability in grain yield across METs. Additionally, we utilized GIS and envirotyping tools to explore associations between environmental features and grain yield, and to define mega-environments. Integrating environmental data into genetic-statistical models facilitated the characterization of G×E interaction patterns and their association with yield performance (Tolhurst et al., 2022). Furthermore, identifying environmental similarities between the experimental network and the TPE can enhance genetic gains through selection (Chaves et al., 2024).

The yield components of soybean are strongly influenced by the environmental effect (Araújo et al., 2024), thus being subject to the G × E interaction (Meyer et al., 2024; Agoyi et al., 2024; Abebe et al., 2024). Over the years, overall performance and stability parameters have been assessed using methods based on analysis of variance (ANOVA) and linear regression. However, several limitations have been identified, such as: (*i*) modeling the genotype effect only as fixed; and (*ii*) the use of balanced data. We fitted a model of the genotype effect as random, employing the factor analytic structure (Piepho, 1997b; Smith et al., 2001a). This approach allows for the estimation of genetic parameters, using the heterogeneous random effect, enabling the evaluation of genetic progress over breeding cycles in various locations, seasons, and different agricultural years (Gogel et al., 2018; Chaves et al., 2023).

The genetic correlation heatmap in **Figure 4** reveals high heterogeneity in genetic variances and low genetic correlations among environments, highlighting the crossover nature of the G × E interaction (Cullis et al., 2010). In other words, as the intensity of the interaction increases, the genetic correlation between pairs of environments decreases. This phenomenon is explained by the disparity in genetic variance values in each environment and the covariance between pairs of environments (Cooper and Delacy, 1994). Heinemann et al. (2022) demonstrated, in the context of crossover G×E interaction, the influence of environmental features on yield components. This can be explained by the direct effect of specific environmental variables on the adaptation of genotypes in METs. Therefore, it becomes crucial to identify environmental factors (climate, soil, spatial trends, among others) and genetic factors influencing the G × E interaction. To achieve this, robust methodologies are necessary to dissect this interaction and enable more precise selection (Kang et al., 1989).

The FA model stands out for its efficiency in handling diverse data structures (Piepho, 1998). This approach is commonly employed in MET, particularly during the stages of cultivar selection and recommendation (Kelly et al., 2007). This becomes possible due to the derivation of orthogonal factors from a set of correlated variables (Cullis et al., 2014). These factors represent linear combinations of the factor loadings associated with each environment, along with the corresponding scores for each cultivar. It is worth noting that the structure of the FA model resembles that of an unstructured covariance matrix but distinguishes itself by its greater parsimony. A study conducted by Chaves et al. (2023) demonstrated the effectiveness and flexibility of FAST in selecting tropical maize genotypes, aiming for overall performance and stability across different locations and seasons. The authors suggested incorporating pedigree or genomic data into the statistical model, applying optimization methods, and using environmental features as strategies to enhance selection estimates.

The evaluation of genotypes with high overall performance and stability can be done through latent regression graphs. Although these graphs provide valuable information, selecting the best cultivars using this methodology can be labor-intensive, as it requires evaluating individualized regression for each genotype. In order to overcome these limitations, Smith and Cullis (2018) proposed FA selection tools, aiming to assess the overall performance and stability of each genotype across the entire dataset. Overall performance is achieved when the loadings of the first factor are positive and rotated, corresponding to the main effects of the genotypes. In this scenario, there is no complex G × E interaction, as the ranking of genotypes remains unchanged across different environments. The RMSD is used to estimate stability by measuring the deviation of each genotype from the line drawn by the latent regression. In this study, weights were assigned to both parameters since, for this specific dataset, productive performance was deemed more critical than stability. Consequently, some studies managed to achieve genetic gains using MET data, employing FAST for cultivar recommendation (Smith and Cullis, 2018; Tolhurst et al., 2019; Bakare et al., 2022).

The environmental and altitudinal characteristics of Malawi and Zambia significantly influence local climatic conditions, vegetation distribution, and land use (**Supplementary Figure S4**). Both countries are situated in high-altitude regions, with Malawi exhibiting altitudes ranging from 500 to 1,500 m, reaching 3,002 m in the Mulanje Mountains (Lancaster, 1980), while Zambia maintains an average altitude between 1,000 and 1,500 m, with Mount Mafinga as its highest peak (2,339 m). These altitudinal variations directly impact temperature regimes, precipitation patterns, and agricultural potential, aligning with previous studies on the influence of topography on African ecosystems. Higher elevations in Malawi are associated with milder temperatures and increased precipitation, which favor diverse vegetation and agricultural systems. In contrast, low-altitude areas, such as regions near Lake Malawi and the Shire Valley, experience warmer and more humid conditions, influencing local biodiversity and crop adaptability. Similarly, Zambia’s elevated plateaus contribute to a moderate climate, reducing temperature extremes and promoting stable precipitation levels (Rawlins and Kalaba, 2020).

The analysis of mega-environments aims to identify target regions or environments with consistent patterns of G×E interaction over multiple years (Yan et al., 2023). When these patterns are stable and repeatable, the target region can be subdivided into sub-regions or mega-environments (Cooper and Hammer, 1996). However, when data are limited to a single year, the mega-environment concept may not be appropriate, as these environments should represent repeatable G×E interaction patterns (Basford and Cooper, 1998). In addition to yield data, incorporating environmental variables such as edaphoclimatic characteristics (elevation, temperature, precipitation, and soil type) can enhance the delineation of mega-environments. These variables provide a more comprehensive understanding of environmental influences on genotype performance, facilitating more precise recommendation strategies for different regions.

In this context, we observed that the variables growing degree days (gdd), mean temperature (Tmean), photosynthetically active radiation use efficiency (fue), seasonal precipitation variability (spv), and temperature range (Tmrange) were the most important factors influencing soybean grain yield in these environments. In tropical and subtropical regions such as Malawi and Zambia, adequate GDD accumulation is essential to ensure that soybean reaches maturity at the appropriate time. Mean temperature directly affects soybean metabolic rates, and excessively high temperatures can induce heat stress, negatively impacting photosynthesis and grain formation. Factors such as light intensity, temperature, and water availability influence fue. In regions with high solar radiation, such as Malawi and Zambia, soybean has the potential for high fue, provided that other factors, such as water and nutrient availability, are not limiting. Irregular precipitation patterns, including severe droughts, can adversely affect soybean development from germination to grain filling. A moderate temperature range is beneficial for soybean, promoting improved carbon assimilation and balanced growth. Understanding the influence of environmental variables on soybean cultivation and modeling the G×E interaction enables the identification of specific adaptations, assisting breeders in decision-making regarding which varieties can have their genetic potential fully exploited (Araújo et al., 2024). Integrating robust statistical models, machine learning techniques (Crossa et al., 2024), and crop growth models (Bustos-Korts et al., 2022) can enhance the accuracy of these recommendations.

## Data Availability

The original contributions presented in the study are included in the article/**Supplementary Material**. Further inquiries can be directed to the corresponding authors.
