# Supplementary material for: Optimizing soybean variety selection for the Pan-African Trial network using factor analytic models and envirotyping
Source: Front Plant Sci. 2025 Jun 6;16:1594736. doi: 10.3389/fpls.2025.1594736 (PMC12179183; doi:10.3389/fpls.2025.1594736)
Supplement: Supplementary file 2 [file DataSheet2.pdf]

Optimizing soybean varieties selection for the Pan-African Trial Network using the analytic factor model and envirotyping

IMMEDIATE

Soybean is a global food crop, and climate change significantly affects seed yield. Therefore, the selection of varieties with high adaptation to target population of environments is imperative in Africa. This study aimed to identify soybean varieties with high overall performance and stability using multi-environment trial data from the Pan-African Soybean Trial Network. Additionally, we sought to determine the environmental factors influencing yield through envirotyping tools. A total of 169 soybean varieties were evaluated across 83 environments in 19 locations in Malawi (47 trials) and 14 locations in Zambia (36 trials). The trials followed a randomized complete block design with three replications. Data for 37 environmental features were obtained from `NASA POWER` and `SoilGrids`. We fitted a factor analytic model (FA) to estimate genotype adaptation across environments. Additionally, we applied an environmental kernel approach to assess environmental similarity. The FA model with four factors provided the best fit, explaining 82.44% and 81.95% of the variance and the ratio of semi-variances, respectively. Approximately 59.6% of the genotype-by-environment interaction was crossover. Varieties V025, V035, and V158 exhibited high yield potential and reliability but displayed moderate stability. Three mega-environments were identified, with growing degree days, mean temperature, and photosynthetically active radiation use efficiency being the most influential features for soybean seed yield. To enhance the identification of variety adaptation in these environments, integrating machine learning models to crop growth modeling is essential to assess the direct influence of environmental features on yield soybean.

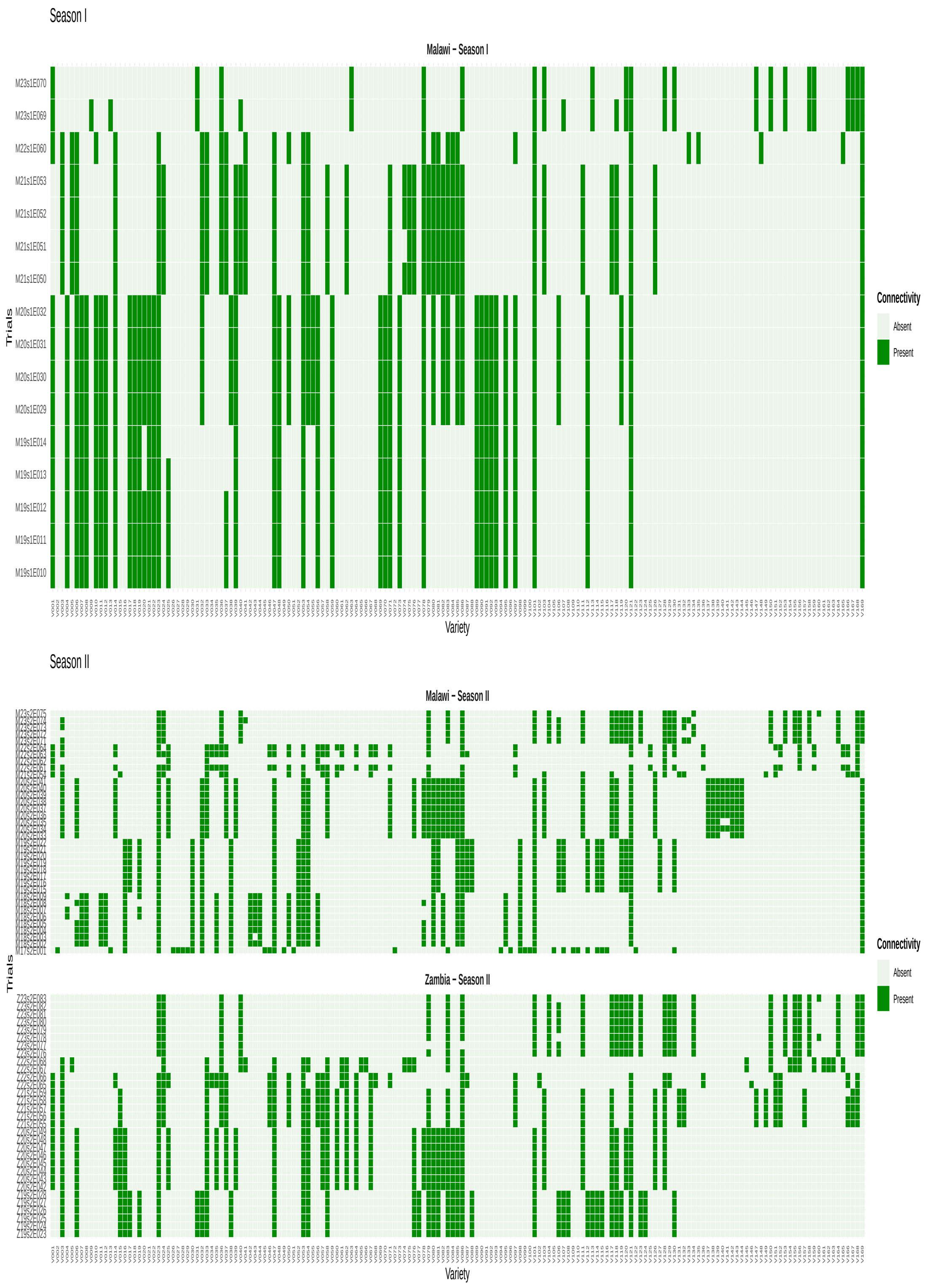

Fig. S1. The figures represent the connectivity between soybean varieties (x-axis) and trials (y-axis). Green-colored pixels indicate the presence of a variety in a trial, while white pixels indicate its absence. The data are divided into two cropping seasons: Malawi includes Seasons I and II, while Zambia includes only Season I. The trials are part of the Pan-African evaluations conducted from 2017/18 to 2023/24.

Table S1. Descriptive statistics of soybean grain yield (kg ha^{-1}) across multi-environment trials conducted in Malawi and Zambia, including geographic coordinates, altitude, and yield distribution metrics (minimum (Min), quartiles (Q1 and Q3), median, maximum (Max), interquartile range (IQR), and overall mean).

Country	Trial	Location	Lat	Long	Alt (m)	Min	Q1	Median	Q3	Max	IQR	Yield (kg ha^{-1})
Malawi	M18s2E002	Karonga	-9.93	33.91	494	291.72	1118.97	1577.93	1826.90	2761.38	707.93	1463.26
Malawi	M18s2E003	Kamatila	-13.97	33.52	1176	1345.00	2531.25	2858.50	3287.25	4419.00	756.00	2884.44
Malawi	M18s2E004	Salima	-13.69	34.25	619	477.00	1361.00	1704.00	2198.25	2989.00	837.25	1794.82
Malawi	M18s2E005	Bvumbwe	-15.92	35.07	1149	882.00	1742.50	2207.50	2760.75	3954.00	1018.25	2257.87
Malawi	M18s2E006	Msoro Road Road	-13.64	32.49	966	899.00	1854.00	2424.00	2707.00	3470.00	853.00	2305.30
Malawi	M18s2E007	Lilongwe	-14.22	33.7	1137	628.00	2459.50	2936.00	3332.00	4814.00	872.50	2905.49
Malawi	M18s2E008	Madisi	-13.42	33.61	1122	799.00	1942.00	2276.00	2727.00	3742.00	785.00	2315.00
Malawi	M18s2E009	Suza	-12.94	33.47	1039	232.00	1249.25	1542.00	1794.25	4382.00	545.00	1682.24
Malawi	M19s1E010	Dedza	-14.26	34.57	511	821.00	2351.00	3420.75	4080.40	5049.80	1729.40	3260.03
Malawi	M19s1E011	Machinga	-15.22	35.49	642	1002.50	1384.40	1599.10	1842.55	3054.10	458.15	1675.41
Malawi	M19s1E012	Water Works	-13.67	32.66	1151	129.50	2076.78	2663.75	3270.55	4793.20	1193.78	2616.72
Malawi	M19s1E013	Kasungu	-13.25	33.37	1061	303.00	1459.50	2041.00	2689.20	4319.10	1229.70	2114.00
Malawi	M19s1E014	Kasungu	-12.56	33.28	1259	711.30	1138.73	1434.35	1815.80	3441.60	677.08	1558.98
Malawi	M19s2E015	Mzimba	-12.12	33.42	1243	265.70	685.33	1484.70	2885.03	4491.00	2199.70	1812.07
Malawi	M19s2E016	Lilongwe	-13.98	33.65	1134	2158.30	2846.83	3246.60	3739.50	4994.70	892.68	3316.54
Malawi	M19s2E017	Salima	-13.67	34.27	599	754.80	1495.08	1977.50	2385.18	2958.70	890.10	1917.09
Malawi	M19s2E018	Thyolo	-15.93	35.07	1149	508.30	1113.58	1444.75	1868.50	2862.80	754.93	1493.68
Malawi	M19s2E019	Zesco Road	-13.66	32.56	1024	295.20	1793.00	2243.25	2912.25	4454.90	1119.25	2337.53
Malawi	M19s2E020	Lilongwe	-13.92	33.68	1099	308.50	2487.40	2976.10	3257.70	3985.00	770.30	2835.91
Malawi	M19s2E021	Madisi	-13.41	33.62	1121	939.30	1867.73	2346.65	3237.20	4531.50	1369.48	2566.67
Malawi	M19s2E022	Suza	-12.94	33.47	1039	1084.50	2441.80	3038.65	3809.53	4927.80	1367.73	3095.43
Malawi	M20s1E029	Ngwerere Road	-15.3	28.3	1189	108.00	2463.00	3248.00	3700.00	5228.00	1237.00	3016.51
Malawi	M20s1E030	Mpongwe	-13.51	28.16	1191	391.00	2219.00	3042.00	3741.00	5149.00	1522.00	2979.18
Malawi	M20s1E031	Chongwe District	-15.34	28.53	1129	1117.96	2959.89	3643.38	4142.47	5282.18	1182.58	3522.29
Malawi	M20s1E032	Chongwe District	-15.39	28.58	1110	165.52	1803.59	2184.48	2724.54	4598.00	920.95	2246.36
Malawi	M20s2E033	Achitende	-10.17	31.23	1358	1814.80	3349.15	3788.40	4209.20	5185.10	860.05	3742.04
Malawi	M20s2E034	Abbsa	-14.44	28.44	1191	704.00	1489.98	2080.40	2559.30	4844.10	1069.33	2078.53
Malawi	M20s2E035	Dedza	-14.27	34.58	511	716.10	2383.13	2842.50	3187.03	4146.20	803.90	2784.83
Malawi	M20s2E036	Machinga	-15.22	35.49	642	614.30	1575.68	1978.30	2364.93	3623.10	789.25	1984.48
Malawi	M20s2E037	Madisi	-13.4	33.63	1121	294.60	1976.88	2471.25	2879.48	4579.60	902.60	2417.01
Malawi	M20s2E038	Suza	-12.94	33.46	1039	1111.30	2295.90	2656.20	3221.70	5280.20	925.80	2793.23
Malawi	M20s2E039	Lilongwe	-14.18	33.8	1150	190.80	708.08	1101.70	1594.78	4405.00	886.70	1243.58
Malawi	M20s2E040	Mzimba	-12.12	33.42	1243	1289.00	2504.10	2884.05	3743.58	4973.80	1239.48	3056.6
Malawi	M20s2E041	Lilongwe	-13.98	33.65	1134	426.50	1789.28	2263.35	2948.20	4154.50	1158.93	2334.36
Malawi	M21s1E050	Salima	-13.67	34.27	599	286.76	2245.67	2840.57	3377.43	4803.78	1131.76	2755.70
Malawi	M21s1E051	Thyolo	-15.93	35.07	1149	225.82	1096.55	1737.93	2600.57	5257.24	1504.02	1956.16
Malawi	M21s1E052	Kambila Village	-13.68	32.73	1177	526.41	2373.10	2967.04	3353.40	4922.37	980.30	2890.62
Malawi	M21s1E053	Lilongwe	-13.95	33.68	1114	292.16	2127.80	2869.94	3815.43	5225.15	1687.63	2925.71
Malawi	M21s2E054	Madisi	-13.41	33.62	1121	319.37	2837.19	3572.10	4248.96	5144.41	1411.78	3440.98
Malawi	M22s1E060	Suza	-12.94	33.47	1039	141.17	1138.11	1478.93	1918.30	3097.20	780.19	1558.82
Malawi	M22s2E061	Ngwerere Road	-15.3	28.3	1203	356.78	1281.33	1670.57	2246.06	4010.28	964.74	1769.84
Malawi	M22s2E062	Chongwe District	-15.29	28.3	1189	3600.92	4032.83	4582.34	4834.94	5037.24	802.11	4410.92
Malawi	M22s2E063	Chilanga District	-15.4	28.08	1218	252.87	1770.11	2662.50	3747.59	5222.50	1977.48	2718.53
Malawi	M22s2E064	Mpongwe	-13.56	28.16	1212	210.00	611.50	1204.00	3256.00	5002.00	2644.50	1874.79
Malawi	M23s1E069	Chongwe District	-15.39	28.58	1110	941.21	2582.36	3165.16	3806.69	5181.61	1224.33	3165.87
Malawi	M23s1E070	Chongwe District	-15.34	28.53	1129	1013.79	2629.89	3034.48	3616.55	5051.72	986.66	3105.58
Malawi	M23s2E071	Kabwe	-14.23	28.29	1198	645.60	2330.66	2969.66	3603.53	4684.60	1272.87	2950.25
Malawi	M23s2E072	MAGOBBO	-15.83	27.91	1019	985.92	1925.79	2199.68	2429.12	3163.81	503.33	2194.54
Malawi	M23s2E073	Dedza	-14.27	34.56	524	133.33	658.34	1000.00	1416.67	2566.67	758.33	1068.47
Malawi	M23s2E074	Machinga	-15.22	35.5	642	2886.44	4002.16	4309.89	4658.85	5283.68	656.69	4287.91
Malawi	M23s2E075	Madisi	-13.4	33.63	1121	254.07	1163.26	1753.70	2326.86	3810.29	1163.60	1743.26
Zambia	Z19s2E023	Suza	-12.94	33.46	1027	605.15	1885.44	2341.20	2627.27	3789.17	741.83	2292.97
Zambia	Z19s2E024	Madisi	-13.4	33.63	1121	1151.98	1963.80	2288.64	2661.30	3818.39	697.51	2312.83
Zambia	Z19s2E025	Chongwe District	-15.34	28.39	1178	314.48	735.40	1050.57	1358.28	2824.14	622.88	1120.91

Continued on next page

Continued from previous page

Country	Trial	Location	Lat	Long	Alt (m)	Min	Q1	Median	Q3	Max	IQR	Yield (kg ha^{-1})
Zambia	Z19s2E026	Ngwerere Road	-15.3	28.31	1203	1224.83	2363.45	2925.00	3335.09	4379.08	971.64	2841.02
Zambia	Z19s2E027	Chilanga District	-15.4	28.08	1218	114.21	337.57	503.28	657.53	1146.93	319.96	523.82
Zambia	Z19s2E028	Chongwe District	-15.34	28.52	1129	247.82	825.88	1401.98	1820.72	3476.51	994.84	1359.29
Zambia	Z20s2E042	Chongwe District	-15.29	28.3	1189	758.63	2204.02	2760.59	3012.80	3879.06	808.78	2612.94
Zambia	Z20s2E043	Suza	-12.94	33.46	1039	566.34	1507.75	1885.03	2308.67	3818.80	800.92	1941.02
Zambia	Z20s2E044	Nthumba	-16.09	34.78	98	1502.07	2911.27	3179.46	3522.93	4712.64	611.66	3171.10
Zambia	Z20s2E045	Kasungu	-12.57	33.4	1224	1492.59	2699.12	3034.97	3370.37	4841.97	671.25	3073.15
Zambia	Z20s2E046	Chinthembwe	-13.41	33.98	1359	1350.78	2007.90	2312.26	2713.31	3739.06	705.41	2351.29
Zambia	Z20s2E047	Chinthembwe	-13.41	33.98	1359	698.61	1382.14	1709.93	2012.09	4126.08	629.95	1732.95
Zambia	Z20s2E048	Ngwerere Road	-15.3	28.31	1203	600.00	2000.00	2400.00	2925.00	4900.00	925.00	2475.00
Zambia	Z20s2E049	Ngwerere Road	-15.3	28.3	1189	920.00	2920.00	3480.00	3890.00	4920.00	970.00	3414.62
Zambia	Z21s2E055	Chilanga District	-15.41	28.08	1218	1550.90	2809.86	3531.95	3972.63	4961.07	1162.77	3406.90
Zambia	Z21s2E056	Chilanga District	-15.41	28.08	1218	103.41	1152.50	1732.70	2441.61	3467.71	1289.11	1798.27
Zambia	Z21s2E057	Crossroads	-14	33.76	1076	464.90	1871.80	2559.60	2807.70	3735.40	935.90	2409.08
Zambia	Z21s2E058	Wimbe	-12.95	33.7	1109	366.00	2679.50	3146.00	3514.00	4624.00	834.50	3045.45
Zambia	Z21s2E059	Maseya	-16.11	34.84	85	350.89	2415.36	3989.35	4664.52	5231.63	2249.16	3439.18
Zambia	Z22s2E065	Msoro Road	-13.63	32.54	990	130.93	742.43	2466.10	3328.25	5291.11	2585.82	2362.54
Zambia	Z22s2E066	Nchalo	-16.25	34.89	70	183.09	884.82	1958.68	3402.86	5238.70	2518.04	2180.86
Zambia	Z22s2E067	Wimbe	-12.95	33.7	1109	1422.93	2202.16	2638.02	3306.73	4424.09	1104.58	2758.71
Zambia	Z22s2E068	Salima	-13.63	34.39	485	1379.31	2263.79	2973.37	3822.61	4961.69	1558.82	3047.16
Zambia	Z23s2E076	Chongwe District	-15.33	28.39	1178	1421.03	3138.39	3661.79	4377.01	5257.93	1238.62	3632.24
Zambia	Z23s2E077	Mkushi District	-13.88	29.11	1158	2326.44	3643.28	4257.47	4721.15	5147.59	1077.87	4170.15
Zambia	Z23s2E078	Ngwerere Road	-15.3	28.31	1203	1259.35	2370.31	3030.05	3337.98	4593.26	967.67	2918.05
Zambia	Z23s2E079	Chilanga District	-15.41	28.08	1218	1636.02	2789.27	3048.28	3505.75	5136.14	716.48	3220.34
Zambia	Z23s2E080	Mpongwe	-13.55	28.06	1211	1267.59	2326.44	3347.13	3802.76	5053.79	1476.32	3219.40
Zambia	Z23s2E081	Chongwe District	-15.34	28.52	1129	286.60	1095.54	1463.54	1746.94	2422.48	651.40	1406.98
Zambia	Z23s2E082	Chongwe District	-15.29	28.3	1189	1659.00	2983.72	3529.40	4028.23	5193.30	1044.51	3505.20
Zambia	Z23s2E083	Kalumbila	-12.42	26.22	1318	491.26	1040.57	1468.81	1821.16	3253.38	780.60	1490.62

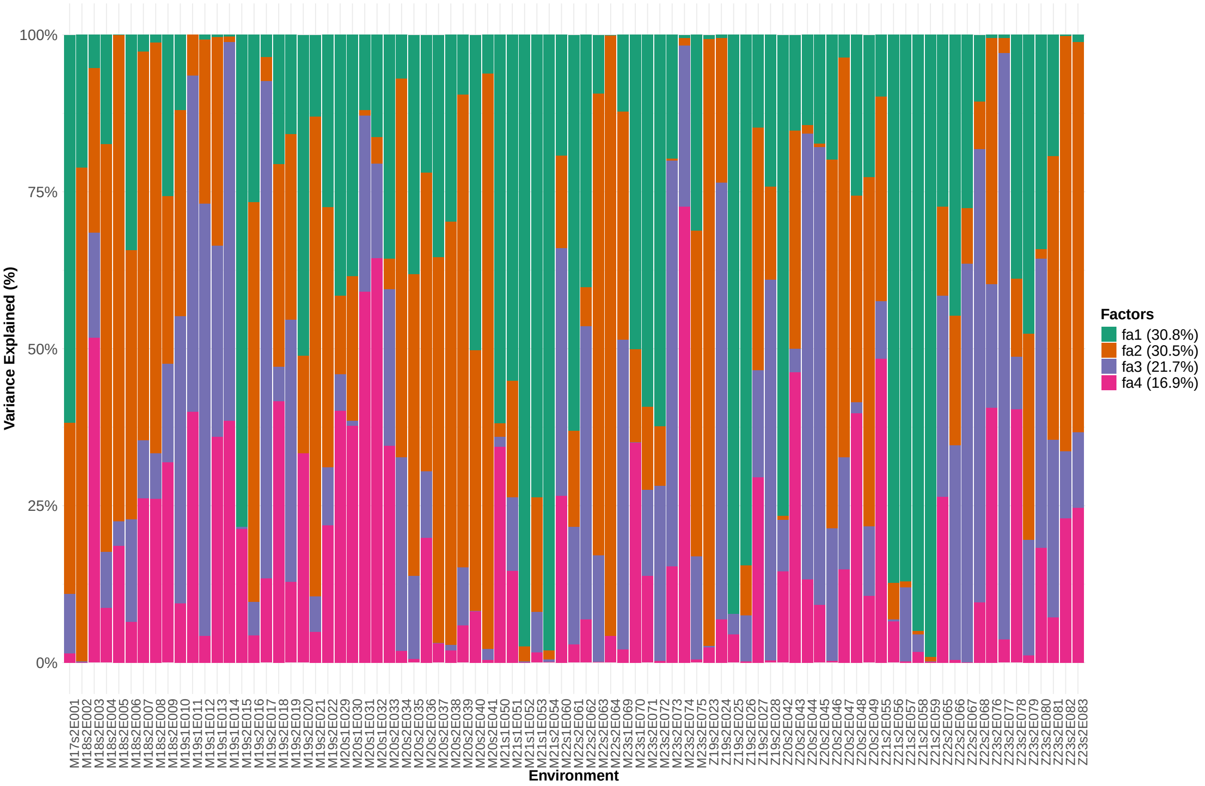

Fig. S2. Representation of the factor analytic (FA) model with a four-factor structure. Each trial is depicted by the proportion of variance accumulated by each factor.

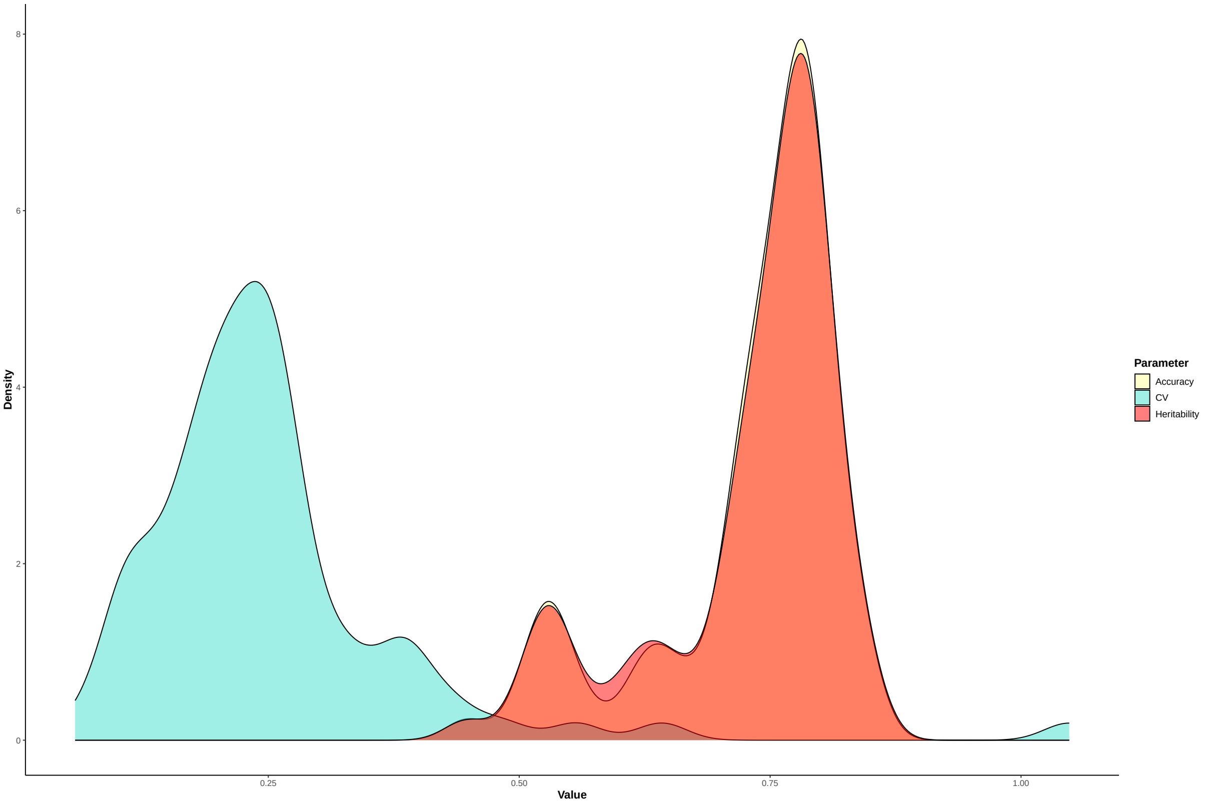

Fig. S3. Density distribution of the parameters accuracy, coefficient of variation (CV), and heritability for soybean variety seed yield. The data were obtained from 83 multi-environment trials conducted under the Pan-African project in the countries of Malawi and Zambia.

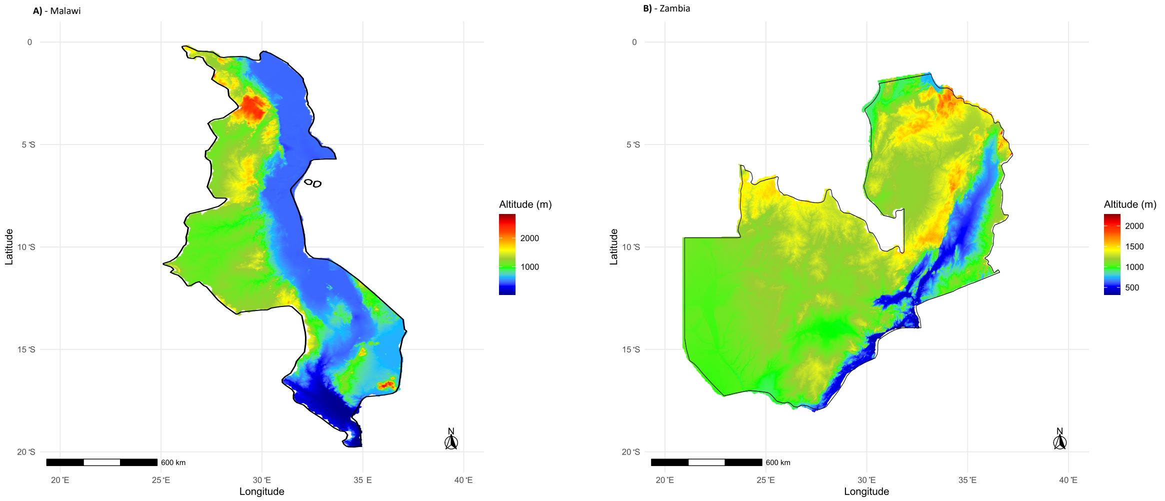

Fig. S4. Map of elevation variation across regions: (A) Malawi and (B) Zambia, highlighting differences in altitude distribution throughout the study areas.
